# Lectin-Mediated Bacterial Modulation by the Intestinal Nematode *Ascaris suum*

**DOI:** 10.3390/ijms22168739

**Published:** 2021-08-14

**Authors:** Ankur Midha, Guillaume Goyette-Desjardins, Felix Goerdeler, Oren Moscovitz, Peter H. Seeberger, Karsten Tedin, Luca D. Bertzbach, Bernd Lepenies, Susanne Hartmann

**Affiliations:** 1 Institute of Immunology, Freie Universität Berlin, 14163 Berlin, Germany; ankur.midha@fu-berlin.de; 2 Institute for Immunology & Research Center for Emerging Infections and Zoonoses (RIZ), University of Veterinary Medicine Hannover, 30559 Hannover, Germany; guillaume.goyette-desjardins@tiho-hannover.de (G.G.-D.); bernd.lepenies@tiho-hannover.de (B.L.); 3 Biomolecular Systems, Max Planck Institute of Colloids and Interfaces, 14476 Potsdam, Germany; felix.goerdeler@mpikg.mpg.de (F.G.); oren.moscovitz@mpikg.mpg.de (O.M.); peter.seeberger@mpikg.mpg.de (P.H.S.); 4 Department of Biology, Chemistry, Pharmacy, Freie Universität Berlin, 14195 Berlin, Germany; 5 Institute of Microbiology and Epizootics, Freie Universität Berlin, 14163 Berlin, Germany; karsten.tedin@fu-berlin.de; 6 Institute of Virology, Freie Universität Berlin, 14163 Berlin, Germany; luca.bertzbach@leibniz-hpi.de; 7 Department of Viral Transformation, Leibniz Institute for Experimental Virology (HPI), 20251 Hamburg, Germany

**Keywords:** *Ascaris*, helminths, intestinal nematode, microbiota, lectin, *Salmonella*, glycan array, C-type lectin, C-type lectin receptor

## Abstract

Ascariasis is a global health problem for humans and animals. Adult *Ascaris* nematodes are long-lived in the host intestine where they interact with host cells as well as members of the microbiota resulting in chronic infections. Nematode interactions with host cells and the microbial environment are prominently mediated by parasite-secreted proteins and peptides possessing immunomodulatory and antimicrobial activities. Previously, we discovered the C-type lectin protein AsCTL-42 in the secreted products of adult *Ascaris* worms. Here we tested recombinant AsCTL-42 for its ability to interact with bacterial and host cells. We found that AsCTL-42 lacks bactericidal activity but neutralized bacterial cells without killing them. Treatment of bacterial cells with AsCTL-42 reduced invasion of intestinal epithelial cells by *Salmonella*. Furthermore, AsCTL-42 interacted with host myeloid C-type lectin receptors. Thus, AsCTL-42 is a parasite protein involved in the triad relationship between *Ascaris*, host cells, and the microbiota.

## 1. Introduction

Intestinal parasitic nematode and other helminth infections are widespread in humans, companion animals, livestock, and wildlife. Ascariasis, caused by *Ascaris lumbricoides* in humans and the closely related *Ascaris suum* in pigs, is one of the most common nematode infections worldwide [1,2]. In humans, ascariasis in children with high worm burdens can lead to malnutrition, developmental deficits, and death [3,4,5]. In pigs, *Ascaris* causes major production losses due to reduced feed conversion and growth rates as well as liver condemnation [6]. Worm burdens vary between individuals, and the majority of the worm burden is carried by a minority of the infected population [7]. The parasite life cycle is thought to follow a similar trajectory in both host species; eggs containing third-stage larvae hatch within hours of ingestion followed by invasion of the cecum and proximal colon [8]. Then, the larvae begin their tissue migration through the liver, reaching the lungs by 6–8 days post-infection (dpi) [9]. The larvae get coughed up and swallowed arriving in the small intestine where they mature into adults, which can reside there for at least 1 year [6]. Pigs are a powerful model for human infectious diseases due to the anatomical, physiological, and genetic similarities between pigs and humans [10], especially in the case of ascariasis where the intestinal tracts and microbiota are more comparable as opposed to widely available mouse models [11]. Furthermore, *Ascaris* is also a zoonotic pathogen, and the porcine gut may represent a reservoir for additional bacterial pathogens such as *Salmonella*, the second most common food-borne pathogen in the European Union [12,13]. Despite the close coexistence of *Ascaris* with numerous microbes, little is known concerning the reciprocal interactions of the nematodes with the microbiota. It has previously been reported that nematode infections lead to changes in intestinal microbial composition [14,15]. One study reported increased alpha diversity in the acute phase at 14 days post-infection (dpi) [14] while another documented decreased diversity in chronically infected pigs at 54 dpi [15]. In both studies, altered microbial compositions were most apparent in the proximal colon, a site with high bacterial loads in contrast to the small intestine where the parasite resides.

Interactions between *Ascaris*, the microbiota, and host cells are mediated in part by the release of excreted and secreted (ES) products [16]. Characterization of the *Ascaris* ES proteome has revealed developmental, life stage-dependent differences in ES content [17]. In addition to structural proteins and proteins involved in molting, motor activity, and metabolism, ES components also contain proteins and peptides with known or predicted antimicrobial and immunomodulatory activities, including antimicrobial peptides, lysozymes, chitinases, cystatins, and lectins [17,18]. Lectins, carbohydrate-binding proteins with numerous functions, are abundant in nematodes [19]. Recently, we discovered several C-type lectin (CTL) domain-containing proteins in the ES of adult *A. suum* nematodes [18]. *A. suum* total ES proteins induce calcium-dependent bacterial agglutination, indicative of CTL-mediated activity [18]. Interestingly, lectin-containing ES from the murine helminth *Heligmosomoides polygyrus* also exhibits calcium-dependent bacterial agglutination [20]. The mammalian lectin RegIIIγ possesses antibacterial activity and maintains segregation between the intestinal microbiota and host epithelium in mice [21]. Furthermore, CTLs are involved in the defense of the free-living nematode *Caenorhabditis elegans* against microbial threats [22,23,24] as well as the maintenance of gut microbiome homeostasis in mosquitoes [25]. Thus, nematode CTLs may defend worms against infection [24] or alternatively may modulate host immune responses [26].

We hypothesized that CTLs from *A. suum* might have microbiota-modulating properties. Therefore, we aimed to determine whether a prominent CTL protein found in *A. suum* ES (hereafter referred to as AsCTL-42) has the potential to modulate the intestinal microbiota. Here, we expressed a recombinant, 42 kilodalton (kDa), signal peptide-containing CTL protein that we had detected in intestine-dwelling adult *A. suum* (UniProt name: C-type lectin domain-containing protein 160, UniProt accession number: F1L7R9) [18]. As host defense molecules can be multi-functional, possessing antimicrobial and immune-modulating activities [27], we tested AsCTL-42 for its effects on the viability of host and bacterial cells, probed for potential binding partners for the protein, and assessed the impact of AsCTL-42 on the invasion of host epithelial cells by the pathogen *Salmonella enterica* subsp. enterica serovar Typhimurium (*S*. Typhimurium).

## 2. Results

### 2.1. Eukaryotic Expression of AsCTL-42

**Figure 1:**
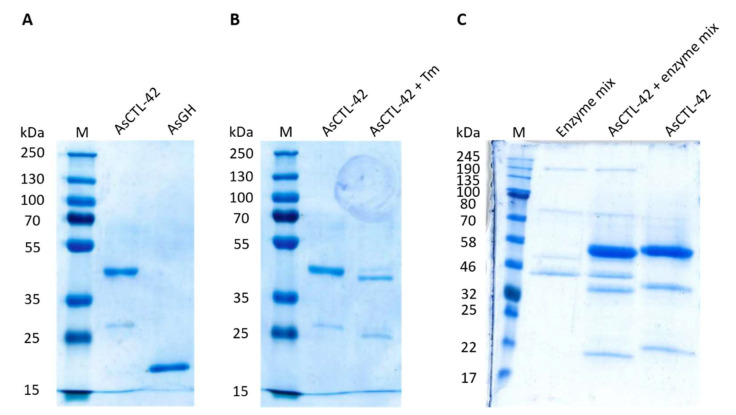
Coomassie-stained SDS-PAGE gels of recombinantly expressed *Ascaris suum* proteins and glycosylation patterns of AsCTL-42. (**A**) 1 µg of protein loaded onto 12% SDS-polyacrylamide gels, stained with Coomassie G-250 dye. (**B**) *Leishmania tarentolae* were cultured in the presence (right; AsCTL-42 + Tm) or absence (left; AsCTL-42) of tunicamycin (10 µg/mL). 1 µg of protein loaded onto 12% SDS-polyacrylamide gels, stained with Coomassie G-250 dye. (**C**) AsCTL-42 was treated with a protein deglycosylation enzyme mixture and the products of this reaction were loaded onto 14% SDS-polyacrylamide gels, stained with Coomassie G-250 dye.

AsCTL-42 and a control protein GH family 25 lysozyme 2 (herein denoted AsGH) were both recombinantly expressed using the eukaryotic *Leishmania tarentolae* expression system (Figure 1A) [28]. For AsCTL-42, we observed a band at a molecular weight between 35 and 55 kDa as well as additional bands of a lower molecular weight. The additional bands were confirmed to be derived from AsCTL-42 by mass spectrometry (Figure S1). To ensure recombinant proteins were free of lipopolysaccharide (LPS) contamination, we used the Endosafe endotoxin testing system as described in the methods. Proteins used in this study were found to have LPS levels below 0.1 ng/mL (less than 1 endotoxin unit per mL). To confirm the presence of post-translational modifications, we cultured *L. tarentolae* in the presence of tunicamycin (10 µg/mL) to inhibit N-glycosylation [29] and observed mobility shifts expected of a glycosylated protein (Figure 1B). To confirm these findings and further assess additional post-translational modifications, AsCTL-42 was treated with a protein deglycosylation enzyme mixture, including PNGase F, O-Glycosidase, α2-3,6,8 Neuraminidase, β1-4 Galactosidase, and β-N-Acetylglucosaminidase. We subjected the products of this reaction to sodium dodecyl sulphate-polyacrylamide gel electrophoresis (SDS-PAGE) and once again observed mobility shifts indicative of glycosylation patterns (Figure 1C). Images of original, uncropped gels are available in the supplementary material (Figure S2).

### 2.2. AsCTL-42 Agglutinates Salmonella

**Figure 2:**
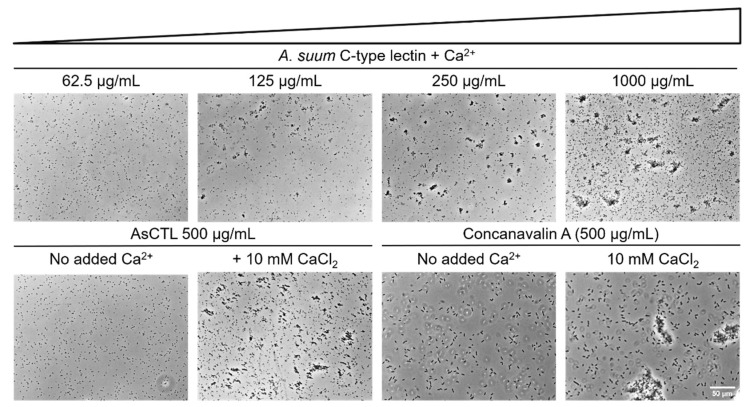
AsCTL-42 agglutinates *Salmonella* in the presence of calcium. Representative images of agglutination of *S*. Typhimurium with increasing concentrations of AsCTL-42. Controls include buffer (tris-buffered saline) without added calcium as well as the C-type lectin concanavalin A with and without added calcium. Bacteria visualized at 400× magnification. Data are representative of two independent experiments performed with independent batches of AsCTL-42.

Nematodes can neutralize microbial threats using CTL proteins [24]. Having shown previously that lectin-containing *A. suum* ES products agglutinate bacteria [18], we sought to determine whether recombinant AsCTL-42 could recapitulate this observation. To test the agglutinating activity of AsCTL-42, we treated *S.* Typhimurium 4/74 with AsCTL-42 in the presence and absence of CaCl_2_ (10 mM) and observed dose- and calcium-dependent agglutinating activity (Figure 2). Interestingly, we also observed reduced motility in agglutinated samples (Supplemental Videos). Thus, recombinant AsCTL-42 is capable of neutralizing potential infectious threats by agglutination.

### 2.3. AsCTL-42 Does Not Inhibit Bacterial Growth

As *Ascaris* nematodes inhabit a rich microbial environment, they need to modulate not only the microbiota of the host’s intestine but also their own microbiota. Microbiota modulation may be achieved via the release of factors with antimicrobial activity. We have previously shown that *A. suum* ES products can inhibit bacterial growth [18]. Amongst the factors we detected in the ES products, we identified several CTL proteins. Lectins have been implicated in shaping the microbiota, in some cases by killing bacteria [25]. We therefore tested whether recombinant AsCTL-42 inhibits the growth of different bacterial strains in comparison to the antimicrobial peptide pexiganan in radial diffusion assays. Treatment with AsCTL-42 did not inhibit the growth of the Gram-positive or Gram-negative bacterial strains that we tested, including *Enterococcus faecium* DSM20477, *Staphylococcus aureus* IMT29828, *Escherichia coli* IMT19224, and *S.* Typhimurium 4/74 (Table 1), all of which are species that can be found in the porcine intestine [30,31,32,33]

### 2.4. AsCTL-42 Does Not Bind to Bacterial Glycans

In order to shed light on the interactome of AsCTL-42, we examined potential binding to different glycan structures via a synthetic glycan array. The glycan array slide contained 140 structurally diverse glycans from bacteria, protozoans, fungi, mammals, and plants as listed in Table S1 [34]. The plant lectin concanavalin A was used as a positive control. However, AsCTL-42 failed to recognize any of the printed structures, even at high protein concentrations (Table S1), while concanavalin A expectedly bound strongly to glycans containing mannose and glucose (Figure S3) [35].

### 2.5. AsCTL-42 Decreases Invasion of Porcine Intestinal Epithelial Cells by Salmonella

**Figure 3:**
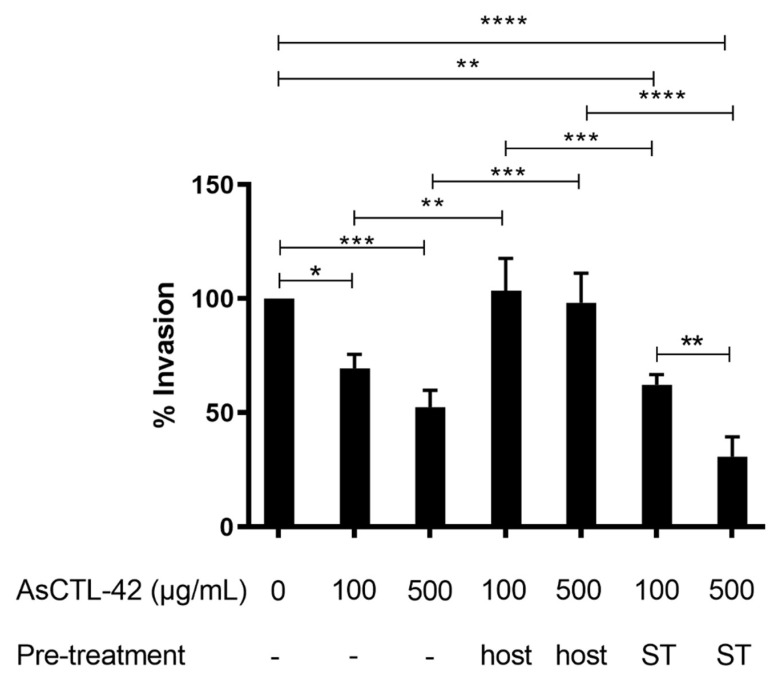
AsCTL-42 impairs porcine intestinal epithelial cell invasion by *Salmonella*. Treatments (AsCTL-42 or PBS as a control) were added to IPEC-J2 cells at the time of infection, or host and bacterial (ST) cells were incubated with treatments for 30 min prior to infection. IPEC-J2 cells were infected by *S.* Typhimurium 4/74 and intracellular CFU were determined. Columns represent mean % invasion (with PBS-treated cells set to 100%) from three independent experiments ± SEM. Significance determined by one-way ANOVA with Tukey’s multiple comparison tests, * *p* < 0.05, ** *p* < 0.005, *** *p* < 0.0005, **** *p* < 0.0001. For clarity, only significant differences have been annotated. All missing comparisons are not statistically significant.

We further assessed the impact of AsCTL-42 treatment on the invasion of intestinal porcine epithelial cells (IPEC-J2) by *Salmonella* using an in vitro invasion assay [36]. We recovered significantly fewer intracellular *Salmonella* from the IPEC-J2 cells in the presence of AsCTL-42 (Figure 3). In order to determine whether the AsCTL-42 reduced bacterial invasion by acting on host or bacterial cells, we performed the experiment by adding the *Ascaris* protein to the culture medium at the same time as the bacteria, or by pre-treating either host or bacterial cells with AsCTL-42 for 30 min prior to infection. We observed a dose-dependent decrease in epithelial cell invasion by *S.* Typhimurium, an effect that was particularly evident when we pre-treated the bacteria prior to infection (Figure 3). Colony-forming unit (CFU) counts from individual experiments can be found in Table S1. Thus, AsCTL-42 is able to reduce the invasion of porcine intestinal epithelial cells by *Salmonella* by acting on bacterial rather than host cells.

### 2.6. AsCTL-42 Does Not Interfere with Host Cell Viability

**Figure 4:**
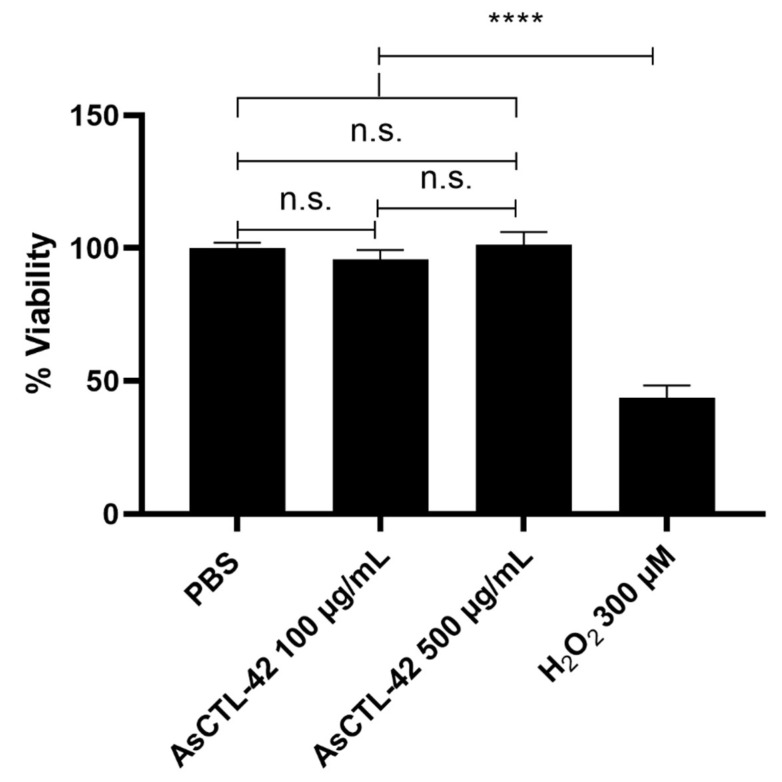
AsCTL-42 treatment does not reduce viability of IPEC-J2 cells. Cells were treated for 24 h with PBS (vehicle control), AsCTL-42 (100 µg/mL, 500 µg/mL), or H_2_O_2_ (300 µM) as a positive control for reduced viability. Cell viability was assessed using the MTT assay. Columns represent mean viability from four independent experiments ± SEM. Significance determined by one-way ANOVA with Tukey’s multiple comparison tests, n.s. = not statistically significant, **** *p* < 0.0001.

As *Ascaris* nematodes dwell in the lumen of the porcine intestine, their ES products may interact with microbes as well as host epithelia. Having determined that AsCTL-42 does not inhibit the growth of various bacterial strains, we sought to determine whether it interferes with host cells. We assessed cell viability of IPEC-J2 cells by the colorimetric MTT assay that involves the conversion of 3-(4,5-dimethylthiazol-2-yl)-2,5-diphenyltetrazolium bromide (MTT) to a formazan product by mitochondrial NAD(P)H-dependent reductases [37]. The formazan product is quantified by absorbance and reflects the viability and metabolic health of the cells. As shown in Figure 4, AsCTL-42 does not inhibit the viability of IPEC-J2 cells.

### 2.7. AsCTL-42 Binds Selected Mammalian C-Type Lectin Receptors

**Figure 5:**
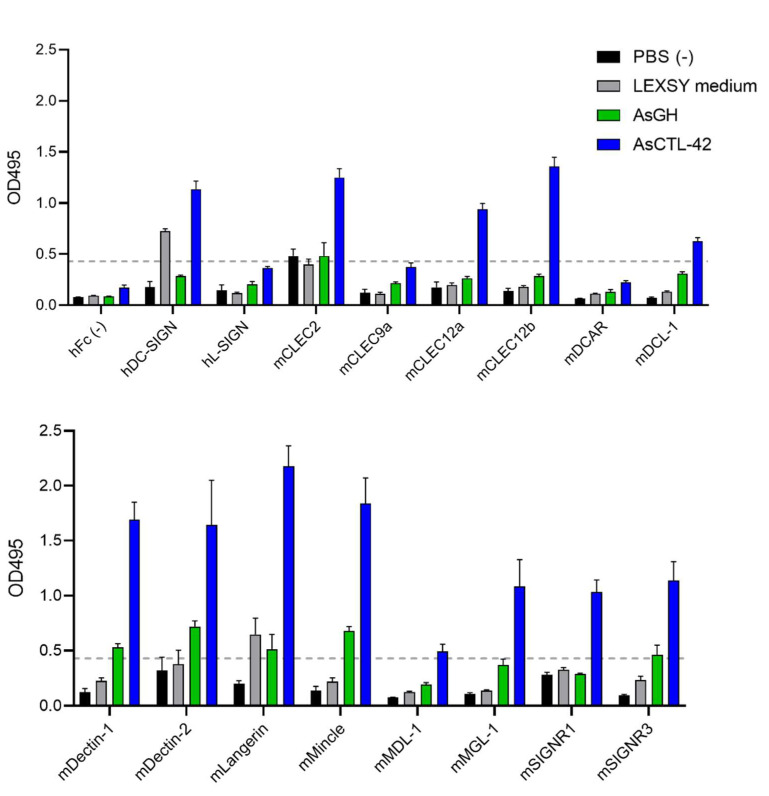
AsCTL-42 binds to selected human (h) and murine (m) C-type lectin receptors. ELISA plates were coated with treatments (0.5 µg) and screened for binding to CLR-hFC fusion proteins. Binding was detected using horseradish peroxidase (HRP)-conjugated goat anti-human IgG to generate absorbance readings at 495 nm with an ELISA plate reader. Spent LEXSY cultivation medium from *L. tarentolae* was included as a control to rule out contribution from *Leishmania* proteins while AsGH was included as an expression system control. Data are presented as mean absorbance readings from three independent experiments ± SEM. The dashed line represents the threshold for CLR binding, defined as four times the average OD values for the hFc control.

In order to assess the potential for AsCTL-42 to bind to host cells, we screened for interactions between AsCTL-42 and C-type lectin receptors (CLR) from humans and mice. We found that AsCTL-42 binds to selected human and murine myeloid CLRs (Figure 5). To verify the specificity of lectin binding, we used another similarly expressed and purified recombinant control protein from Ascaris, AsGH, which did not demonstrate strong binding to myeloid CLRs (Figure 5). To further rule out non-specific effects due to the expression system, we included an *L. tarentolae* medium that did not exhibit notable binding compared to AsCTL-42 (Figure 5).

**Figure 6:**
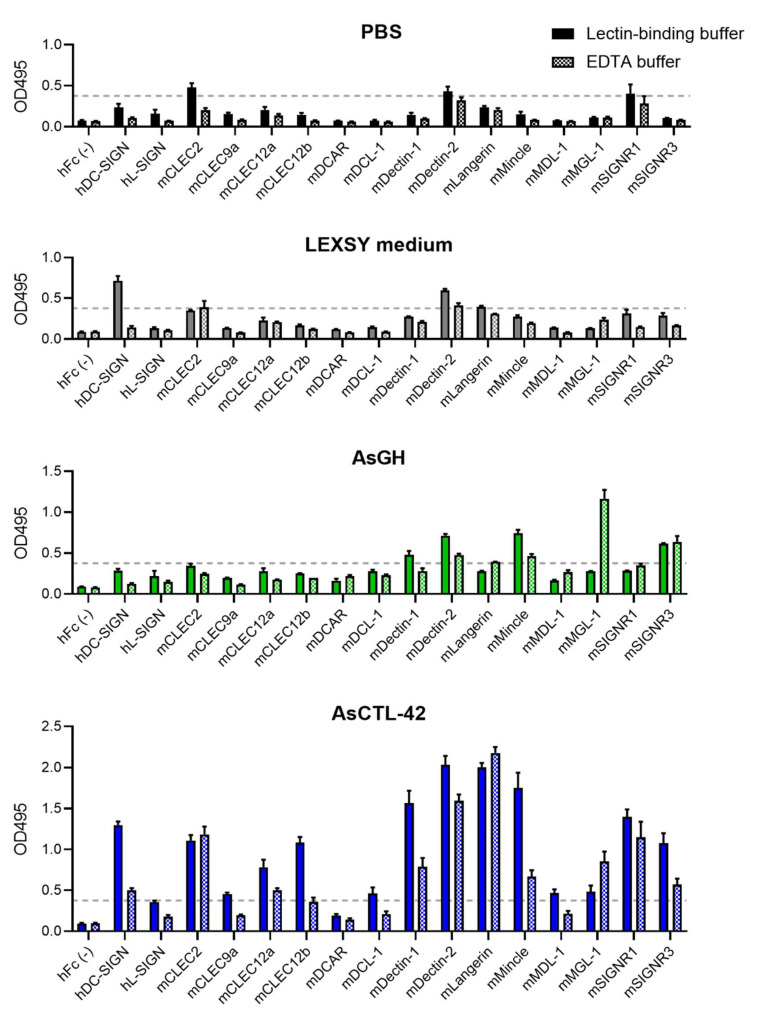
Binding of AsCTL-42 to C-type lectin receptors is calcium-dependent. ELISA plates were coated with treatments (0.5 µg) and screened for binding to CLR-hFC fusion proteins in the presence of calcium-containing lectin binding buffer (solid bars) or EDTA buffer (checkered bars). Binding was detected using horseradish peroxidase (HRP)-conjugated goat anti-human IgG to generate absorbance readings at 495 nm with an ELISA plate reader. Spent LEXSY cultivation medium from *L. tarentolae* was included as a control to rule out contribution from *Leishmania* proteins while AsGH was included as an expression system control. Data are presented as average absorbance readings from three independent experiments ± SEM. The dashed line represents the threshold for CLR binding, defined as four times the average OD values for the hFc control.

Interestingly, AsCTL-42-CLR binding appeared to be calcium-dependent, as binding tended to decrease in the presence of EDTA (Figure 6).

Particularly prominent binding was observed for Dectin-1, Dectin-2, Langerin, and Mincle. These data indicate that AsCTL-42 has the potential to interact with host cells and may have immunomodulating activities via CLRs. The corresponding porcine CLRs can be found in Table 2.

## 3. Discussion

Intestinal nematodes inhabit a rich microbial environment. In addition to confronting host immunity, these organisms must contend with microbial cohabitants including potential pathogens. Studies of these multi-lateral interactions have demonstrated that helminths can sense host microbes and also rely on them for proper development, infectivity, and fecundity [20,38,39]. Parasite-driven immune responses can alter the production of host defense molecules and mucin resulting in alterations to the microbiota [40]. Furthermore, interactions between bacteria and helminths can be mutually beneficial as was shown for *Lactobacillus taiwanensis* and *H. polygyrus* where both species promote each other in the murine gut [41]. While numerous studies have documented microbiome alterations associated with nematode infections, the underlying mechanisms can be quite complex and difficult to decipher as interactions between host, microbes, and parasites can be direct and indirect as well as multi-directional.

Nematode ES products include a cocktail of proteins and peptides possessing antimicrobial and immunomodulatory activities [18,20,42]. Lectin domain-containing proteins, including CTLs and galectins, were prominent in the ES products of intestine-dwelling adult *Ascaris* worms [18]. Lectins are best known for their glycan-binding properties and perform multiple biological functions. The *A. suum* genome encodes at least 78 lectin domain-containing sequences, including 36 CTLs [19]. Secreted lectins may be cytotoxic, as was shown for the CTL CEL-1 from the sea cucumber *Pseudocnus echinatus* (formerly *Cucumaria echinata*), which exhibits cytotoxicity against numerous cell lines [43]. In this study, we demonstrated that the secreted lectin AsCTL-42 from *A. suum* does not directly impact the viability of host or bacterial cells. There was no detectable influence of AsCTL-42 on host cell viability using the porcine intestinal epithelial cell line IPEC-J2 (Figure 4) that is representative of the host cells in the immediate vicinity of *Ascaris.* Lectins are also under investigation for their diverse antimicrobial activities [44]; however, we did not detect any influence on the viability of different bacterial strains in this study (Table 1). Unlike the bactericidal mammalian lectin RegIIIγ, nematode lectins have thus far not shown bactericidal activity. This is consistent with our data showing that AsCTL-42 may play a non-lethal role in modulating microbial populations, as has also been observed for lectins from *C. elegans* where selected CTLs released by the nematode in response to bacterial exposure are able to bind the bacteria without killing them [22,23,24].

Although our data show that AsCTL-42 is not bactericidal, it exhibits a non-toxic antimicrobial activity. We detected calcium-dependent bacterial agglutination by AsCTL-42 (Figure 2). Our previous work showed that ES products from *A. suum* and *H. polygyrus* agglutinate bacteria in a calcium-dependent manner [18,20]. Interestingly, CTLs are upregulated in response to microbial threats in *C. elegans* [22,23] and recombinant *clec-39* and *-49* bind bacteria without killing them in a calcium-independent manner [24]. Although we did not identify glycan binding partners in the glycan arrays, the presence of a lectin domain does not assure sugar-binding. Previously, glycan array screening using *clec-39* and *-49* from *C. elegans* did not reveal carbohydrate binding partners [24]. Furthermore, CTLs may also bind to non-glycan ligands [45], and only eight of the 36 CTLs encoded in the *A. suum* genome are predicted to bind carbohydrate ligands by hidden Markov modeling [19]. The agglutinating activity we detected confirms that AsCTL-42 does indeed interact with bacterial cells. Together, these observations suggest that secreted nematode lectins may neutralize bacterial threats.

In addition to interactions with microbial cells, we also demonstrated the potential for AsCTL-42 to interact with mammalian cells. As myeloid CLRs can sense microbes such as Gram-negative bacteria, fungi, *Mycobacterium* spp., trematodes, and viruses [46,47], their modulation has implications for intestinal microbial communities. We found that AsCTL-42 interacts with selected human and murine myeloid CLRs (Figure 5). Interestingly, these interactions were calcium-dependent, as the addition of EDTA tended to reduce binding (Figure 6). CLR modulation has been documented for different helminth species. DC-SIGN is a receptor for egg antigens from the trematode *Schistosoma mansoni* [48] while Dectin-1 on macrophages was found to be a target of immunomodulation by the sheep liver fluke *Fasciola hepatica* [49]. While we did not assess porcine CLRs, the porcine parasite *A. suum* and the human parasite *A. lumbricoides* are both capable of infecting pigs and humans [12]. Notably, paleoparasitological and genetic evidence indicate that *A. suum* and *A. lumbricoides* are the same species [50]. Thus, interactions between *A. suum* and human receptors are insightful for both human and porcine ascariasis. In addition, human, murine, and porcine CLRs overlap considerably (Table 2). Our observations suggest a potential for *Ascaris* lectins to directly influence host myeloid cells but downstream consequences for host microbiota modulation remain to be determined.

Having determined that AsCTL-42 can interact with both bacteria and host cells, we sought to determine the functional consequences of such interactions. It has been shown previously that helminth infections can modulate immune responses against intracellular pathogens [51]. Thus far, there have been no reports of how *Ascaris* might influence immune responses against *Salmonella* even though both pathogens are prevalent in pigs, are of considerable zoonotic importance, and there exists an association between high *Ascaris* exposure and *Salmonella* prevalence in pigs [52]. Thus, we studied the relationship between *Ascaris*, *Salmonella*, and host cells using an in vitro porcine epithelial cell invasion assay. We found that AsCTL-42 reduced the invasion of intestinal epithelial IPEC-J2 cells by *Salmonella* by acting on the bacteria rather than on host cells (Figure 3). Pre-treating host cells prior to infection did not reduce epithelial cell invasion while pre-treating bacterial cells did; hence, we attribute our observations to agglutination and the reduced motility of *Salmonella* in the presence of AsCTL-42. Interestingly, a previous study found that *H. polygyrus* infection altered the metabolomic environment of the murine intestine and that these metabolomic alterations promoted coinfection of mice with *Salmonella* [53]. We have demonstrated the potential for one particular lectin protein to decrease epithelial cell invasion, though *Ascaris* ES products contain numerous other factors, including metabolites. Notably, *A. suum* can produce short-chain fatty acids [54] that can have mixed effects on *Salmonella* virulence, growth, and motility [55,56,57]. Though our data point to potentially meaningful interactions between these two pathogens, further study is warranted to determine the outcomes and mechanisms underlying interactions between *Ascaris* and *Salmonella* in vivo.

A potential limitation of our work is posed by the high concentrations of AsCTL-42 used in some of the experiments. While we focused on one particular CTL in vitro, it is likely that multiple lectins may act synergistically in vivo and we have previously identified several lectin domain-containing proteins in *A. suum* ES products [18]. Furthermore, helminths including *Ascaris* are frequently found aggregated together in the host intestine [58,59] where sexually mature adult worms must be in close proximity to mate. *A. suum,* also referred to as the ‘large roundworm’, is indeed quite large—individual worms can weigh up to 7 g and measure up to 30 cm in length [60]. In severe cases, this aggregation can obstruct the intestine [6]. Notably, parasite burdens of well over 100 worms per host have been observed in humans and pigs [7,15]. We speculate that in a natural system, numerous worms aggregating in the rather narrow confines of the jejunum could collectively produce lectin-containing ES products in considerable concentrations. Thus, we consider the concentrations used herein as insightful, particularly in the case of individuals with high worm burdens, due to the specific microenvironment that *Ascaris* adults are found in and the composition of their ES products.

In summary, our findings suggest that secreted CTLs considerably aid the establishment of *A. suum* in the porcine intestine. Others have speculated on a role for helminth CTLs in parasite–host interactions [61,62]. Previous studies have also pointed to host CLRs as important modulators of the host immune response against helminths [48,49]. Here, we provide support for these observations having shown that AsCTL-42 can interact with myeloid CLRs. We have identified several potential binding candidates, which warrant further study. Furthermore, we have demonstrated a role for AsCTL-42 in directly modulating microbes through its interactions with *Salmonella*. Future studies should be carried out to elucidate the mechanistic underpinnings of AsCTL-42–bacterial interactions, in particular to determine the bacterial binding partner of AsCTL-42. Further investigation may place lectins amongst a handful of other well-studied helminth immunomodulators, such as cystatins, helminth defense molecules, and transforming growth factor beta mimic proteins [63,64,65]. Considered in context, the multiple lectins produced by *Ascaris* would have evolved to ensure parasite survival within the host, perhaps by binding to multiple targets.

## 4. Materials and Methods

### 4.1. Recombinant Expression of AsCTL-42 and Protein Analysis

AsCTL-42 and AsGH were both recombinantly expressed using the eukaryotic *Leishmania* expression system (LEXSY; Jena Bioscience, Jena, Germany) as described previously [28,66]. The nucleotide sequences of AsCTL-42 and AsGH without their specific signal sequences were cloned into the pLEXSY-sat2 plasmid of the LEXSYcon2 Expression kit. Following manufacturer’s instructions, a monoclonal LEXSY cell strain expressing and secreting the desired target protein with a hexa-histidine tag was developed. Purification of the protein was performed via affinity chromatography using HisTrap™excel columns and the ÄKTA™ pure chromatography system (GE Healthcare Bio-Science AB, Uppsala, Sweden) using imidazole as a competitive eluent in a non-denaturing protocol. Purified proteins were dialyzed against PBS, sterile filtered, and protein concentrations were determined using the Pierce™ BCA Protein Assay Kit (Pierce Biotechnology, Rockford, IL, USA). LPS contamination was assessed using Endosafe^®^ PTS cartridges (Charles River Laboratories, Charleston, VA, USA). Protein mass was assessed by SDS-PAGE on 12% agarose gels followed by Coomassie staining. We confirmed the identity of the observed bands by LC-MS/MS analysis. Briefly, bands were removed from the gel and protein was retrieved by in-gel tryptic digestion followed by reconstitution in 0.1% trifluoroacetic acid in 2:98 acetonitrile/water. LC-MS/MS analysis and protein identifications of the peptides were performed on an Ultimate 3000 RSLCnano system online coupled to an Orbitrap Q Excative Plus mass spectrometer (Thermo Fisher Scientific, Waltham, MA, USA) followed by database searching using Mascot software version 2.6.1 (Matrix Science Ltd., London, UK) against an internal database (359 sequences), SwissProt 2017_11 (556,196 sequences), and a contaminant database (247 sequences) as described previously [15].

### 4.2. Bacterial Strains

The bacterial strains used to evaluate antibacterial activity of AsCTL-42 in the radial diffusion assay included: *Enterococcus faecium* DSM20477 (kindly provided by Dr. Markus Heimesaat, Institute of Microbiology, Infectious Diseases and Immunology, Charité—Universitätsmedizin Berlin), *Escherichia coli* IMT19224, *Staphylococcus aureus* IMT29828, and *Salmonella enterica* subsp. enterica serovar Typhimurium 4/74, all obtained from the strain collection of the Institute of Microbiology and Epizootics, Freie Universität Berlin. *S*. Typhimurium 4/74 was used to assess agglutinating activity of AsCTL-42 and in epithelial cell invasion assays.

### 4.3. Radial Diffusion Assay

Bacterial growth inhibition activity of AsCTL-42 was assessed using the radial diffusion assay as described previously [18,20]. Overnight bacterial cultures were diluted 1:100 in Mueller–Hinton broth (Carl Roth, Karlsruhe, Germany) and incubated at 37 °C with shaking at 250 rpm until reaching an optical density of 0.3–0.4 at 600 nm. Bacteria were then centrifuged at 880× *g* for 10 min at 4 °C, washed once, and resuspended with cold sodium phosphate buffer (100 mM, pH 7.4). Bacteria were resuspended in 50 °C sterile underlay agar (10 mM sodium phosphate, 1% (*v*/*v*) Mueller-Hinton broth, 1.5% (*w*/*v*) agar) at 4 × 10^5^ colony forming units (CFU) per mL. Fifteen milliliters of underlay agar were poured into 120 mm square petri dishes. After the agar solidified, evenly spaced wells (5 mm) were formed using the blunt end of P10 pipet tips. Treatments were added to the wells (5 µL/well) and the plates incubated at 37 °C for 3 h before being overlaid with 15 mL of double-strength Mueller–Hinton agar (4.2% (*w*/*v*) Mueller–Hinton broth, 1.5% (*w*/*v*) agar). Petri dishes were incubated at 37 °C for 18 h and growth inhibition zones around each well were measured. Growth inhibition is represented as the diameter of the inhibition zone (mm) beyond the well. PBS and the antimicrobial peptide pexiganan (kindly provided by Prof. Jens Rolff, Institute of Biology, Freie Universität Berlin) were used as negative and positive controls, respectively.

### 4.4. Cell Culture and Growth Conditions

Porcine intestinal epithelial cells (IPEC-J2 cell line) were cultured as monolayers in DMEM/Ham’s F-12 (1:1) medium supplemented with 10% fetal calf serum (both from PAN-Biotech, Aidenbach, Germany) under standard tissue culture conditions (37 °C, 5% CO_2_). Experiments were performed within five passages after seeding the original frozen stocks. *Salmonella* invasion assays were performed in the presence of 5 mM CaCl_2_.

### 4.5. Cell Viability Testing

For cell viability assays, IPEC-J2 cells were seeded at 5 × 10^3^ cells/well in 96-well tissue culture plates and grown until ~80% confluence prior to treatment. Cells were incubated with PBS (vehicle control), different concentrations of AsCTL-42 diluted in PBS, or 300 µM H_2_O_2_ (positive control [67]) for 24 h. Viability was assessed using the MTT cell proliferation kit (Roche Diagnostics, Mannheim, Germany) according to the manufacturer’s instructions. Briefly, after 24 h treatments, 10 µL of MTT reagent [3-(4,5-dimethylthiazol-2-yl)-2,5-diphenyltetrazolium bromide) were added to each well for 4 h followed by overnight solubilization of formazan crystals in the incubator with 100 µL of solubilization solution (10% SDS in 0.01 M HCl). Absorbance was measured in a Biotek Synergy H1 Hybrid microplate reader at 570 nm. Cell viability was calculated by normalizing treatment groups to PBS-treated cells as 100% viability controls. Statistical analyses were performed using GraphPad Prism 9.0.1 to conduct a one-way ANOVA followed by Tukey’s multiple comparison tests. *p*-values less than 0.05 were considered significant.

### 4.6. Glycan Array

The array contained 140 different synthetic glycans (0.2 mM), printed in the lab on *N*-hydroxyl succinimide ester-activated slides as described previously (Table S1) [34]. Glycans were immobilized on slides using a piezoelectric spotting device (S3; Scienion, Berlin, Germany) in a pattern of 16 individual subarrays. After 24 h in a humid chamber at room temperature, the slides were quenched using 50 mM aminoethanol solution (pH 9) for 1 h at 50 °C and a final ddH_2_O wash before storage. Next, 16-well microplate holders were assembled onto the slides and each well was blocked with 100 µL of HEPES buffer (50 mM HEPES pH 7.2, 5 mM CaCl_2_, 5 mM MgCl_2_) with 1% BSA for 1 h. After washing blocked wells with HEPES buffer without BSA, 75 µL of AsCTL-42 at different concentrations (5, 10, 50, 100, 200 µg/mL) and concanavalin A fluorescein (25 µg/mL, Vector Labs, Burlingame, USA) were added to each well followed by 1 h incubation. Each concentration was tested in duplicates. The wells were washed three times with HEPES buffer + 0.05% Tween and incubated with 75 µL of 6xHis tag monoclonal antibody FITC (1:200, Invitrogen) for 1 h in a dark, humidified chamber. The wells were washed once with HEPES buffer + 0.05% Tween. Then the microplate holder was removed and the whole slide was washed twice with the HEPES buffer + 0.05% Tween and once with the HEPES buffer without detergent. The slide was dried by centrifugation (300× *g*, 3 min) and directly scanned using a Glycan Array Scanner Axon GenePix^®^ 4300A (Molecular Devices, San Jose, CA, USA). Results were analyzed using GenePix Pro7 (Molecular Devices).

### 4.7. C-Type Lectin Receptor Screening

The generation of the CLR-hFc fusion protein library was described previously [34,68,69,70]. Treatments were diluted to 10 µg/mL in PBS, then 50 µL (0.5 µg) were added to each well of a medium binding half-area 96-well ELISA plate (Greiner Bio-One, Kremsmünster, Austria). Plates were left overnight at 4 °C. The next day, plates were washed three times with PBS containing 0.05% (*v*/*v*) Tween 20 (PBST) then blocked with the addition of 150 µL of PBS containing 1% (*w*/*v*) BSA for 2 h. After washing, 50 µL (0.25 µg) of CLR-hFc fusion proteins, diluted at 5 µg/mL in either lectin-binding buffer (50 mM HEPES, 5 mM CaCl_2_, 5 mM MgCl_2_ (pH 7.4)) or EDTA buffer (50 mM HEPES, 10 mM EDTA (pH 7.4)), was added to each well for 1 h. After washing, the plates were incubated for 1 h with 50 µL of a horseradish peroxidase (HRP)-conjugated goat anti-human IgG (Fcγ fragment specific; Jackson Immunoresearch West Grove, USA) diluted 1:5000 in PBST containing 1% BSA. The enzyme reaction was developed by the addition of 50 µL of o-phenylenediamine dihydrochloride (OPD; Thermo Fisher Scientific), stopped by the addition of 50 µL of 2.5 M H_2_SO_4_, and the absorbance was read at 495 nm with an ELISA plate reader. Spent LEXSY cultivation medium from *L. tarentolae* was included to rule out contribution from *Leishmania* proteins. AsGH was included as an expression system control. Potential binding with the specified CLR was defined as an OD value greater than four times the OD of hFc negative controls.

### 4.8. Agglutination Assay

Agglutinating activity of AsCTL-42 was assessed as described previously [18,20], using *S.* Typhimurium strain 4/74. Bacteria grown in Luria–Bertani (LB) medium were collected at mid-logarithmic phase by centrifugation at 880× *g* for 5 min. They were then washed and re-suspended in tris-buffered saline (50 mM Tris-HCl, 150 mM NaCl, pH 7.5) at approximately 10^9^ cells/mL. Twenty microliters of bacterial suspension were mixed with 20 µL treatments (diluted in TBS) with or without added calcium (10 mM CaCl_2_) and incubated for 1 h at room temperature on a glass slide. Concanavalin A from *Canavalia ensiformis* (Sigma-Aldrich, St. Louis, MO, USA) was included as a positive control. Samples were visualized and photographed using the 40× objective (final 400× magnification) on a Leica DM750 microscope equipped with an ICC50HD digital camera (Leica Microsystems, Wetzlar, Germany).

### 4.9. Salmonella Invasion Assay

For invasion assays, IPEC-J2 cells were grown to a density of ~5 × 10^4^ cells/well in 48-well tissue culture plates and infected at multiplicities of infection (moi) of 1–5. *Salmonella* was grown in an LB medium with aeration at 37 °C to late log/early stationary phase (optical density of 2–3 at 600 nm) and collected from 1 mL of culture suspension by centrifugation and resuspended in 1 mL LB medium. Optical density was determined, and dilutions were made to provide the final moi. Treatments were either added at the time of infection or separately to pre-treat host and bacterial cells 30 min prior to infection, as indicated. Cells were infected for 30 min, then the culture medium was changed and supplemented with 50 µg/mL gentamicin (PAN-Biotech) to kill extracellular bacteria and the cells were incubated for 2 h. Cells were then washed twice with PBS and lysed by the addition of 0.1% (*v*/*v*) Triton X-100 in distilled water. Dilutions of the resulting lysates were plated on LB agar plates for the determination of intracellular CFU. Invasion was determined by the ratio of intracellular CFU to the CFU of the original infecting bacterial suspension. Invasion was calculated by normalizing treatment groups to PBS-treated cells as 100% invasion controls. Statistical analyses were performed using GraphPad Prism 9.0.1 to conduct a 1-way ANOVA followed by Tukey’s multiple comparison tests. *p*-values less than 0.05 were considered significant.

## Figures and Tables

**Table 1:** Bacterial growth inhibition activity ^1^ of AsCTL-42 in the radial diffusion assay.

	*E. faecium* DSM20477	*S. aureus* IMT29828	*E. coli* IMT19224	*S.* Typhimurium 4/74
AsCTL-42 (1 mg/mL)	-	-	-	-
Pexiganan (1.25 µg/mL)	5.0	12.0	11.0	11.0
PBS	-	-	-	-

^1^ Activity reported as diameter of inhibition zone (mm) produced by treatments (*n* = 3 independent batches of AsCTL protein). “-” indicates no detectable activity. Data are representative of two independent experiments.

**Table 2:** Human and murine C-type lectin receptors tested in this study and their corresponding receptors in pigs ^1^.

Human (h) or Murine (m) Protein	Human or Murine Gene	Corresponding Porcine Protein	Corresponding Porcine Gene
hDC-SIGN	*CD209/CLEC4L*	CD209	*CD209*
hL-SIGN	*CD209L/CLEC4M*	CD209	*CD209*
mCLEC2	*Clec1b*	CLEC1b	*CLEC1B*
mCLEC9a	*Clec9a*	CLEC9a	*CLEC9A*
mCLEC12a	*Clec12a*	CLEC12a	*CLEC12A*
mCLEC12b	*Clec12b*	CLEC12b	*CLEC12B*
mDCAR	*Clec4b1*	CLEC4A	*CLEC4A*
mDCL-1	*Clec2i*	CLEC2D	*CLEC2D*
mDectin-1	*Clec7a*	CLEC7A	*CLEC7A*
mDectin-2	*Clec6a*	No corresponding protein	No corresponding gene
mLangerin	*Cd207*	CLEC4K	*CD207*
mMincle	*Clec4e*	CLEC4E	*CLEC4E*
mMDL-1	*Clec5a*	CLEC5A	*CLEC5A*
mMGL-1	*Clec10a*	Asialoglycoprotein receptor 1	*ASGR1*
mSIGNR1	*Cd209b*	CD209	*CD209*
mSIGNR3	*Cd209d*	CD209	*CD209*

^1^ Corresponding gene and protein names obtained using protein BLAST functions on Uniprot (https://www.uniprot.org, accessed on 28 June 2021) and National Center for Biotechnology Information (https://blast.ncbi.nlm.nih.gov/Blast.cgi, accessed on 28 June 2021). Where appropriate, the closest match in BLAST searches were assigned as the corresponding porcine proteins and genes.

## Supplementary Materials

The following are available online at https://www.mdpi.com/article/10.3390/ijms22168739/s1.

## References

[1] World Health Organization Fact Sheet: Soil-Transmitted Helminth Infections. http://www.who.int/mediacentre/factsheets/fs366/en/.

[2] Katakam K.K., Thamsborg S.M., Dalsgaard A., Kyvsgaard N.C., Mejer H. (2016). Environmental Contamination and Transmission of Ascaris Suum in Danish Organic Pig Farms. Parasites Vectors.

[3] Jardim-Botelho A., Raff S., Rodrigues R.D., Hoffman H.J., Diemert D.J., Corrêa-Oliveira R., Bethony J.M., Gazzinelli M.F. (2008). Hookworm, Ascaris Lumbricoides Infection and Polyparasitism Associated with Poor Cognitive Performance in Brazilian Schoolchildren. Trop. Med. Int. Health.

[4] Saathoff E., Olsen A., Kvalsvig J.D., Appleton C.C. (2004). Patterns of Geohelminth Infection, Impact of Albendazole Treatment and Re-Infection after Treatment in Schoolchildren from Rural KwaZulu-Natal/South-Africa. BMC Infect. Dis..

[5] Yentur Doni N., Yildiz Zeyrek F., Simsek Z., Gurses G., Sahin İ. (2015). Risk Factors and Relationship Between Intestinal Parasites and the Growth Retardation and Psychomotor Development Delays of Children in Şanlıurfa, Turkey. Türkiye Parazitolojii Derg..

[6] Dold C., Holland C.V. (2011). Ascaris and Ascariasis. Microbes Infect..

[7] Holland C.V., Asaolu S.O., Crompton D.W., Stoddart R.C., Macdonald R., Torimiro S.E. (1989). The Epidemiology of Ascaris Lumbricoides and Other Soil-Transmitted Helminths in Primary School Children from Ile-Ife, Nigeria. Parasitology.

[8] Murrell K.D., Eriksen L., Nansen P., Slotved H.C., Rasmussen T. (1997). Ascaris Suum: A Revision of Its Early Migratory Path and Implications for Human Ascariasis. J. Parasitol..

[9] Roepstorff A., Eriksen L., Slotved H.C., Nansen P. (1997). Experimental Ascaris Suum Infection in the Pig: Worm Population Kinetics Following Single Inoculations with Three Doses of Infective Eggs. Parasitology.

[10] Meurens F., Summerfield A., Nauwynck H., Saif L., Gerdts V. (2012). The Pig: A Model for Human Infectious Diseases. Trends Microbiol..

[11] Heinritz S.N., Mosenthin R., Weiss E. (2013). Use of Pigs as a Potential Model for Research into Dietary Modulation of the Human Gut Microbiota. Nutr. Res. Rev..

[12] Crompton D.W. (2001). Ascaris and Ascariasis. Adv. Parasitol..

[13] Bonardi S. (2017). Salmonella in the Pork Production Chain and Its Impact on Human Health in the European Union. Epidemiol. Infect..

[14] Williams A.R., Krych L., Ahmad H.F., Nejsum P., Skovgaard K., Nielsen D.S., Thamsborg S.M. (2017). A Polyphenol-Enriched Diet and Ascaris Suum Infection Modulate Mucosal Immune Responses and Gut Microbiota Composition in Pigs. PLoS ONE.

[15] Wang Y., Liu F., Urban J.F., Paerewijck O., Geldhof P., Li R.W. (2019). Ascaris Suum Infection Was Associated with a Worm-Independent Reduction in Microbial Diversity and Altered Metabolic Potential in the Porcine Gut Microbiome. Int. J. Parasitol..

[16] Midha A., Ebner F., Schlosser-Brandenburg J., Rausch S., Hartmann S. (2021). Trilateral Relationship: Ascaris, Microbiota, and Host Cells. Trends Parasitol..

[17] Wang T., Van Steendam K., Dhaenens M., Vlaminck J., Deforce D., Jex A.R., Gasser R.B., Geldhof P. (2013). Proteomic Analysis of the Excretory-Secretory Products from Larval Stages of Ascaris Suum Reveals High Abundance of Glycosyl Hydrolases. PLoS Negl. Trop. Dis..

[18] Midha A., Janek K., Niewienda A., Henklein P., Guenther S., Serra D.O., Schlosser J., Hengge R., Hartmann S. (2018). The Intestinal Roundworm Ascaris Suum Releases Antimicrobial Factors Which Interfere With Bacterial Growth and Biofilm Formation. Front. Cell Infect. Microbiol..

[19] Bauters L., Naalden D., Gheysen G. (2017). The Distribution of Lectins across the Phylum Nematoda: A Genome-Wide Search. Int. J. Mol. Sci..

[20] Rausch S., Midha A., Kuhring M., Affinass N., Radonic A., Kühl A.A., Bleich A., Renard B.Y., Hartmann S. (2018). Parasitic Nematodes Exert Antimicrobial Activity and Benefit From Microbiota-Driven Support for Host Immune Regulation. Front. Immunol..

[21] Vaishnava S., Yamamoto M., Severson K.M., Ruhn K.A., Yu X., Koren O., Ley R., Wakeland E.K., Hooper L.V. (2011). The Antibacterial Lectin RegIIIgamma Promotes the Spatial Segregation of Microbiota and Host in the Intestine. Science.

[22] Mallo G.V., Kurz C.L., Couillault C., Pujol N., Granjeaud S., Kohara Y., Ewbank J.J. (2002). Inducible Antibacterial Defense System in C. Elegans. Curr. Biol..

[23] O’Rourke D., Baban D., Demidova M., Mott R., Hodgkin J. (2006). Genomic Clusters, Putative Pathogen Recognition Molecules, and Antimicrobial Genes Are Induced by Infection of C. Elegans with M. Nematophilum. Genome Res..

[24] Miltsch S.M., Seeberger P.H., Lepenies B. (2014). The C-Type Lectin-like Domain Containing Proteins Clec-39 and Clec-49 Are Crucial for Caenorhabditis Elegans Immunity against Serratia Marcescens Infection. Dev. Comp. Immunol..

[25] Pang X., Xiao X., Liu Y., Zhang R., Liu J., Liu Q., Wang P., Cheng G. (2016). Mosquito C-Type Lectins Maintain Gut Microbiome Homeostasis. Nat. Microbiol..

[26] Hewitson J.P., Grainger J.R., Maizels R.M. (2009). Helminth Immunoregulation: The Role of Parasite Secreted Proteins in Modulating Host Immunity. Mol. Biochem. Parasitol..

[27] Hancock R.E.W., Haney E.F., Gill E.E. (2016). The Immunology of Host Defence Peptides: Beyond Antimicrobial Activity. Nat. Rev. Immunol..

[28] Breitling R., Klingner S., Callewaert N., Pietrucha R., Geyer A., Ehrlich G., Hartung R., Müller A., Contreras R., Beverley S.M. (2002). Non-Pathogenic Trypanosomatid Protozoa as a Platform for Protein Research and Production. Protein Expr. Purif..

[29] Jin S.-P., Chung J.H. (2018). Inhibition of N-Glycosylation by Tunicamycin Attenuates Cell–Cell Adhesion via Impaired Desmosome Formation in Normal Human Epidermal Keratinocytes. Biosci. Rep..

[30] Hammerum A.M. (2012). Enterococci of Animal Origin and Their Significance for Public Health. Clin. Microbiol. Infect..

[31] Khanna T., Friendship R., Dewey C., Weese J.S. (2008). Methicillin Resistant Staphylococcus Aureus Colonization in Pigs and Pig Farmers. Vet. Microbiol..

[32] Luppi A. (2017). Swine Enteric Colibacillosis: Diagnosis, Therapy and Antimicrobial Resistance. Porc. Health Manag..

[33] Casanova-Higes A., Marín-Alcalá C.M., Andrés-Barranco S., Cebollada-Solanas A., Alvarez J., Mainar-Jaime R.C. (2019). Weaned Piglets: Another Factor to Be Considered for the Control of Salmonella Infection in Breeding Pig Farms. Vet. Res..

[34] Geissner A., Reinhardt A., Rademacher C., Johannssen T., Monteiro J., Lepenies B., Thépaut M., Fieschi F., Mrázková J., Wimmerova M. (2019). Microbe-Focused Glycan Array Screening Platform. Proc. Natl. Acad. Sci. USA.

[35] Cummings R.D., Darvill A.G., Etzler M.E., Hahn M.G., Varki A., Cummings R.D., Esko J.D., Stanley P., Hart G.W., Aebi M., Darvill A.G., Kinoshita T., Packer N.H., Prestegard J.H. (2015). Glycan-Recognizing Probes as Tools. Essentials of Glycobiology.

[36] Singh V., Finke-Isami J., Hopper-Chidlaw A.C., Schwerk P., Thompson A., Tedin K. (2017). Salmonella Co-Opts Host Cell Chaperone-Mediated Autophagy for Intracellular Growth *. J. Biol. Chem..

[37] Stockert J.C., Horobin R.W., Colombo L.L., Blázquez-Castro A. (2018). Tetrazolium Salts and Formazan Products in Cell Biology: Viability Assessment, Fluorescence Imaging, and Labeling Perspectives. Acta Histochem..

[38] Chang J., Wescott R.B. (1972). Infectivity, Fecundity, and Survival of Nematospiroides Dubius in Gnotobiotic Mice. Exp. Parasitol..

[39] Wescott R.B., Todd A.C. (1964). A Comparison of the Development of Nippostrongylus Brasiliensis in Germ-Free and Conventional Mice. J. Parasitol..

[40] Fricke W.F., Song Y., Wang A.-J., Smith A., Grinchuk V., Mongodin E., Pei C., Ma B., Lu N., Urban J.F. (2015). Type 2 Immunity-Dependent Reduction of Segmented Filamentous Bacteria in Mice Infected with the Helminthic Parasite Nippostrongylus Brasiliensis. Microbiome.

[41] Reynolds L.A., Smith K.A., Filbey K.J., Harcus Y., Hewitson J.P., Redpath S.A., Valdez Y., Yebra M.J., Finlay B.B., Maizels R.M. (2014). Commensal-Pathogen Interactions in the Intestinal Tract: Lactobacilli Promote Infection with, and Are Promoted by, Helminth Parasites. Gut Microbes.

[42] Ebner F., Hepworth M.R., Rausch S., Janek K., Niewienda A., Kühl A., Henklein P., Lucius R., Hamelmann E., Hartmann S. (2014). Therapeutic Potential of Larval Excretory/Secretory Proteins of the Pig Whipworm Trichuris Suis in Allergic Disease. Allergy.

[43] Kuramoto T., Uzuyama H., Hatakeyama T., Tamura T., Nakashima T., Yamaguchi K., Oda T. (2005). Cytotoxicity of a GalNAc-Specific C-Type Lectin CEL-I toward Various Cell Lines. J. Biochem..

[44] Coelho L.C.B.B., dos Santos Silva P.M., de Oliveira W.F., de Moura M.C., Pontual E.V., Gomes F.S., Paiva P.M.G., Napoleão T.H., dos Santos Correia M.T. (2018). Lectins as Antimicrobial Agents. J. Appl. Microbiol..

[45] Mitchell D.E., Gibson M.I. (2015). Latent Ice Recrystallization Inhibition Activity in Nonantifreeze Proteins: Ca2+-Activated Plant Lectins and Cation-Activated Antimicrobial Peptides. Biomacromolecules.

[46] Zhang P., Snyder S., Feng P., Azadi P., Zhang S., Bulgheresi S., Sanderson K.E., He J., Klena J., Chen T. (2006). Role of N-Acetylglucosamine within Core Lipopolysaccharide of Several Species of Gram-Negative Bacteria in Targeting the DC-SIGN (CD209). J. Immunol..

[47] Lindenwald D.L., Lepenies B. (2020). C-Type Lectins in Veterinary Species: Recent Advancements and Applications. Int. J. Mol. Sci..

[48] van Die I., van Vliet S.J., Nyame A.K., Cummings R.D., Bank C.M.C., Appelmelk B., Geijtenbeek T.B.H., van Kooyk Y. (2003). The Dendritic Cell–Specific C-Type Lectin DC-SIGN Is a Receptor for Schistosoma Mansoni Egg Antigens and Recognizes the Glycan Antigen Lewis x. Glycobiology.

[49] Guasconi L., Serradell M.C., Garro A.P., Iacobelli L., Masih D.T. (2011). C-Type Lectins on Macrophages Participate in the Immunomodulatory Response to Fasciola Hepatica Products. Immunology.

[50] Leles D., Gardner S.L., Reinhard K., Iñiguez A., Araujo A. (2012). Are Ascaris Lumbricoides and Ascaris Suum a Single Species?. Parasites Vectors.

[51] Ahmed N., French T., Rausch S., Kühl A., Hemminger K., Dunay I.R., Steinfelder S., Hartmann S. (2017). Toxoplasma Co-Infection Prevents Th2 Differentiation and Leads to a Helminth-Specific Th1 Response. Front. Cell Infect. Microbiol..

[52] van der Wolf P.J., Wolbers W.B., Elbers A.R., van der Heijden H.M., Koppen J.M., Hunneman W.A., van Schie F.W., Tielen M.J. (2001). Herd Level Husbandry Factors Associated with the Serological Salmonella Prevalence in Finishing Pig Herds in The Netherlands. Vet. Microbiol..

[53] Reynolds L.A., Redpath S.A., Yurist-Doutsch S., Gill N., Brown E.M., van der Heijden J., Brosschot T.P., Han J., Marshall N.C., Woodward S.E. (2017). Enteric Helminths Promote Salmonella Co-Infection by Altering the Intestinal Metabolome. J. Infect. Dis..

[54] Tielens A.G.M., van Grinsven K.W.A., Henze K., van Hellemond J.J., Martin W. (2010). Acetate Formation in the Energy Metabolism of Parasitic Helminths and Protists. Int. J. Parasitol..

[55] Lawhon S.D., Maurer R., Suyemoto M., Altier C. (2002). Intestinal Short-Chain Fatty Acids Alter *Salmonella* Typhimurium Invasion Gene Expression and Virulence through BarA/SirA. Mol. Microbiol..

[56] Jacobson A., Lam L., Rajendram M., Tamburini F., Honeycutt J., Pham T., Treuren W.V., Pruss K., Stabler S.R., Lugo K. (2018). A Gut Commensal-Produced Metabolite Mediates Colonization Resistance to Salmonella Infection. Cell Host Microbe.

[57] Lamas A., Regal P., Vázquez B., Cepeda A., Franco C.M. (2019). Short Chain Fatty Acids Commonly Produced by Gut Microbiota Influence Salmonella Enterica Motility, Biofilm Formation, and Gene Expression. Antibiotics.

[58] José M.V., Ruiz A., Bobadilla J.R. (1997). Prevalence of Infection, Mean Worm Burden and Degree of Worm Aggregation as Determinants of Prevalence of Disease Due to Intestinal Helminths. Arch. Med. Res..

[59] Newey S., Shaw D.J., Kirby A., Montieth P., Hudson P.J., Thirgood S.J. (2005). Prevalence, Intensity and Aggregation of Intestinal Parasites in Mountain Hares and Their Potential Impact on Population Dynamics. Int. J. Parasitol..

[60] Walker M., Hall A., Basáñez M.-G., Holland C. (2013). Chapter 7—Ascaris lumbricoides: New Epidemiological Insights and Mathematical Approaches. Ascaris: The Neglected Parasite.

[61] Loukas A., Maizels R.M. (2000). Helminth C-Type Lectins and Host-Parasite Interactions. Parasitol. Today.

[62] Harcus Y., Nicoll G., Murray J., Filbey K., Gomez-Escobar N., Maizels R.M. (2009). C-Type Lectins from the Nematode Parasites Heligmosomoides Polygyrus and Nippostrongylus Brasiliensis. Parasitol. Int..

[63] Caraballo L., Zakzuk J., Acevedo N. (2021). Helminth-Derived Cystatins: The Immunomodulatory Properties of an Ascaris Lumbricoides Cystatin. Parasitology.

[64] Robinson M.W., Donnelly S., Hutchinson A.T., To J., Taylor N.L., Norton R.S., Perugini M.A., Dalton J.P. (2011). A Family of Helminth Molecules That Modulate Innate Cell Responses via Molecular Mimicry of Host Antimicrobial Peptides. PLoS Pathog..

[65] Smyth D.J., Harcus Y., White M.P.J., Gregory W.F., Nahler J., Stephens I., Toke-Bjolgerud E., Hewitson J.P., Ivens A., McSorley H.J. (2018). TGF-β Mimic Proteins Form an Extended Gene Family in the Murine Parasite Heligmosomoides Polygyrus. Int. J. Parasitol..

[66] Venugopal G., Mueller M., Hartmann S., Steinfelder S. (2017). Differential Immunomodulation in Human Monocytes versus Macrophages by Filarial Cystatin. PLoS ONE.

[67] Jiang S.-H., Shang L., Xue L.-X., Ding W., Chen S., Ma R.-F., Huang J.-F., Xiong K. (2014). The Effect and Underlying Mechanism of Timosaponin B-II on RGC-5 Necroptosis Induced by Hydrogen Peroxide. BMC Complement. Altern. Med..

[68] Maglinao M., Eriksson M., Schlegel M.K., Zimmermann S., Johannssen T., Götze S., Seeberger P.H., Lepenies B. (2014). A Platform to Screen for C-Type Lectin Receptor-Binding Carbohydrates and Their Potential for Cell-Specific Targeting and Immune Modulation. J. Control. Release.

[69] Mayer S., Moeller R., Monteiro J.T., Ellrott K., Josenhans C., Lepenies B. (2018). C-Type Lectin Receptor (CLR)–Fc Fusion Proteins As Tools to Screen for Novel CLR/Bacteria Interactions: An Exemplary Study on Preselected Campylobacter Jejuni Isolates. Front. Immunol..

[70] Prado Acosta M., Goyette-Desjardins G., Scheffel J., Dudeck A., Ruland J., Lepenies B. (2021). S-Layer From Lactobacillus Brevis Modulates Antigen-Presenting Cell Functions via the Mincle-Syk-Card9 Axis. Front. Immunol..

