# Supplementary material for: Lectin-Mediated Bacterial Modulation by the Intestinal Nematode Ascaris suum
Source: Int J Mol Sci. 2021 Aug 14;22(16):8739. doi: 10.3390/ijms22168739 (PMC8395819; doi:10.3390/ijms22168739)
Supplement: Supplementary file 1 [file ijms-22-08739-s001.zip › Table S1_CFU.pdf]

Table S1. AsCTL-42 impairs porcine epithelial cell invasion by *Salmonella*.[1]

	**Experiment 1**		**Experiment 2**		**Experiment 3**	
	**Inoculum (Mean CFU)**	**Number of Epithelial Cells/Well**	**Inoculum (Mean CFU)**	**Number of Epithelial Cells/Well**	**Inoculum (Mean CFU)**	**Number of Epithelial Cells/Well**
	179,000	50,000	82,000	50,000	121,667	50,000
	**Multiplicity of Infection (moi)** = 4		**Multiplicity of Infection (moi)** = 2		**Multiplicity of Infection (moi)** = 2	
**Treatment**	**Mean (CFU/mL)**	**% Invasion Rel. to Control**	**Mean (CFU/mL)**	**% Invasion Rel. to Control**	**Mean (CFU/mL)**	**% Invasion Rel. to Control**
AsCTL-42 0 µg/mL	12,600	100	48,133	100	9,800	100
AsCTL-42 100 µg/mL	8,467	67	36,733	76	6,333	65
AsCTL-42 500 µg/mL	6,607	48	29,333	61	4,733	48
AsCTL-42 100 µg/mL + IPEC pre-treatment	14,533	115	51,733	107	8,600	88
AsCTL-42 500 µg/mL + IPEC pre-treatment	14,000	111	47,400	98	8,333	85
AsCTL-42 100 µg/mL + ST pre-treatment	8,400	67	29,922	62	5,667	58
AsCTL-42 500 µg/mL + ST pre-treatment	2,800	22	19,000	39	3,000	31

[1]Data presented here as CFU counts from the individual experiments collated in Figure 3.
