# Supplementary material for: Lectin-Mediated Bacterial Modulation by the Intestinal Nematode Ascaris suum
Source: Int J Mol Sci. 2021 Aug 14;22(16):8739. doi: 10.3390/ijms22168739 (PMC8395819; doi:10.3390/ijms22168739)
Supplement: Supplementary file 1 [file ijms-22-08739-s001.zip › Table S2.pdf]

**Table S2.** Synthetic glycans tested for AsCTL-42-glycan binding in glycan array[1].		
**Glycan**	Microbial origin (if applicable)	Mean Fluorescence Intensity (MFI) for ConA[2]
Neu5Ac(a2-6)Gal(b1-4)GlcNAc(b1-3)Gal(b1-4)Glc(b1-1)aminohexanol		337,5
Neu5Ac(a2-3)Gal(b1-3)GlcNAc(b1-3)Gal(b1-4)Glc(b1-1)aminohexanol		224
Fuc(a1-3)[Neu5Ac(a2-3)Gal(b1-4)]GlcNAc(b1-3)Gal(b1-4)Glc(b1-1)aminohexanol		0
Neu5Ac(a2-6)Gal(b1-4)Glc(b1-1)aminohexanol		188,5
Neu5Ac(a2-3)Gal(b1-4)Glc(b1-1)aminohexanol		251,5
Neu5Ac(a2-6)Gal(b1-4)GlcNAc-6-sulfate(b1-1)aminohexanol		0
Gal(b1-4)Glc(b1-1)aminohexano		1116,5
Gal(b1-4)GlcNAc-6-sulfate(b1-1)aminohexanol		0
Araf(a1-5)Araf(a1-1)aminopentanol	*Mycobacterium tuberculosis*	1628,5
Araf(a1-5)Araf(a1-3)[Araf(a1-5)Araf(a1-5)]Araf(a1-5)Araf(a1-1)aminopentanol	*Mycobacterium tubercolosis*	1807
Araf(a1-3)[Araf(a1-5)]Araf(a1-1)aminopentanol	*Mycobacterium tuberculosis*	1815,5
Araf(a1-5)Araf(a1-5)Araf(a1-5)Araf(a1-5)Araf(a1-5)Araf(a1-5)aminopentanol	*Mycobacterium tuberculosis*	1890
Col(a1-3)[Col(a1-6)]Glc(a1-4)Gal(a1-3)GlcNAc(b1-1)aminopentanol	*Escherichia coli* O111	367
ManNAc(b1-3)FucNAc(a1-3)GalNAc(a1-4)Gal(a1-1)aminopentanol	*Streptococcus pneumoniae*	208,5
GalNAc(a1-4)Gal(a1-1)aminopentanol	*Streptococcus pneumoniae*	1649

GalNAc(b1-4)Gal(a1-1)aminopentanol		1492,5
FucNAc(a1-3)GalNAc(a1-4)Gal(a1-1)aminopentanol	*Streptococcus pneumoniae*	244
FucNAc(b1-3)GalNAc(a1-4)Gal(a1-1)aminopentanol		250
GalNAc(b1-1)aminoethanol		2051,5
FucNAc(a1-1)aminopentanol		622
Man(a1-2)Man(a1-2)[Gal(b1-4)]Man(a1-1)aminopentanol	*Leishmania donovani*	12566
Man(a1-2)Man(a1-2)Man(a1-1)aminopentanol	*Leishmania donovani*	10525,5
Gal(b1-4)Man(a1-1)aminopentanol	*Leishmania chagasi*	1940
Man(a1-2)Man(a1-1)aminopentanol		16754
Glc(b1-1)aminoethanol		0
GlcNAc(a1-2)Hep(a1-3)Hep(a1-5)Kdo(a2-1)aminopentanol	*Neisseria meningitidis*	491
Hep(a1-3)Hep(a1-5)Kdo(a2-1)aminopentanol	*Neisseria meningitidis*	539,5
Hep(a1-3)Hep(a1-5)[L-Ara4N(b1-8)]Kdo(a2-1)aminopentanol	*Proteus spp.*	216,5
Hep(a1-7)Hep(a1-3)Hep(a1-5)Kdo(a2-1)aminopentanol	*Yersinia pesti*	168,5
Hep(a1-2)Hep(a1-3)Hep(a1-5)Kdo(a2-1)aminopentanol	*Haemophilus influenzae*	695
Hep(a1-5)Kdo(a2-1)aminopentanol		879
Hep(a1-7)Hep(a1-3)Hep(a1-1)aminopentanol	*Yersinia pesti*	437,5
Kdo(a2-8)Kdo(a2-4)Kdo(a2-1)aminopentanol	*Chlamydia spp.*	551
Kdo(a2-1)aminopentanol		103,5
Hep(a1-1)aminopentanol		866,5
Glc(b1-1)aminopentanol		344
D-FucNAc(b1-1)aminopentanol		620,5
FucNAc(b1-1)aminopentanol		296

Glc(b1-3)D-FucNAc(b1-1)aminopentanol	*Pseudomonas aeruginosa*	367,5
Glc(b1-3)FucNAc(b1-1)aminopentanol	*Pseudomonas aeruginosa*	635,5
Gal(b1-3)GalNAc(a1-1)aminopentanol		1080,5
Fuc(a1-3)[Gal(b1-4)]GlcNAc(b1-1)aminopentanol		14,5
Neu5Ac(a2-6)GalNAc(a1-1)aminopentanol		118,5
Gal(b1-4)[Gal(b1-4)Glc(b1-6)]GlcNAc(b1-1)aminopentanol	*Streptococcus pneumoniae*	555
Fuc(a1-3)[Fuc(a1-2)Gal(b1-4)]GlcNAc(b1-1)aminopentanol		457
Gal(b1-3)[Fuc(a1-4)]GlcNAc(b1-1)aminopentanol		341
Fuc(a1-2)Gal(b1-3)[Fuc(a1-4)]GlcNAc(b1-1)aminopentanol		0
Gal-2,3-Pyruvate(a1-1)aminopentanol (mixture of R/S pyruvate)	*Streptococcus pneumoniae*	239,5
Gal(a1-3)Gal(b1-4)Glc(b1-1)aminopentanol		248,5
Gal(a1-3)Gal(b1-4)GlcNAc(b1-1)aminopentanol		117,5
Gal(a1-3)Gal(b1-4)GlcNAc(b1-3)Gal(b1-4)Glc(b1-1)aminopentanol		0
Gal(b1-4)GlcNAc(b1-3)Gal(b1-4)Glc(b1-1)aminopentanol	*Streptococcus pneumoniae*	0
Fuc(a1-2)Gal(b1-3)GlcNAc(b1-3)Gal(b1-4)Glc(b1-1)aminopentanol		1767
6 Gal(b1-3)GlcNAc(b1-3)Gal(b1-4)Glc(b1-1)aminopentanol		303,5
Rha(a1-1)aminopentanol		0
Rha(a1-3)Glc(b1-1)aminopentanol		1504,5
Glc(a1-2)Glc(a1-1)aminopentanol	*Clostridium difficile*	5359
Glc(b1-4)Glc(a1-2)Glc(a1-1)aminopentanol	*Clostridium difficile*	3637
Rha(a1-3)Glc(b1-4)Glc(a1-1)aminopentanol	*Clostridium difficile*	636,5

Gal(b1-3)GalNAc(b1-3)Gal(a1-4)Gal(b1-4)Glc(b1-1)aminopentanol		1205,5
Neu5Ac(a2-8)Neu5Ac(a2-3)[GalNAc(b1-4)]Gal(b1-4)Glc(b1-1)aminopentanol		162
Gal(a1-4)Gal(b1-4)Glc(b1-1)aminopentanol		487,5
GalNAc(a1-1)AminoLinker2		989
Fuc(a1-3)[Gal(b1-4)]GlcNAc(b1-1)AminoLinker2		735
GlcNAc(a1-2)Hep(a1-3)Hep(a1-1)aminopentanol	*Neisseria meningitidis*	558,5
Hep(a1-3)Hep(a1-1)aminopentanol		267,5
Gal(b1-4)Glc(b1-1)aminopentanol		0
GalNAc(b1-4)Gal(b1-4)Glc(b1-1)aminopentanol		1193,5
Neu5Ac(a2-3)Gal(b1-4)Glc(b1-1)aminopentanol		127,5
GalNAc-4-sulfate(b1-1)aminopentanol		19
IdoA-2,4-disulfate(a1-1)aminopentanol		528
IdoA(a1-3)GalNAc-4-sulfate(b1-1)aminopentanol		237
IdoA-2-sulfate(a1-3)GalNAc-4-sulfate(b1-1)aminopentanol		262
IdoA(a1-3)GalNAc(b1-1)aminopentanol		210,5
GlcA(b1-4)Glc(b1-3)GlcA(b1-4)Glc(b1-1)aminoethanol	*Streptococcus pneumoniae*	378
Glc(b1-3)GlcA(b1-4)Glc(b1-1)aminoethanol	*Streptococcus pneumoniae*	1456
GalNAc(a1-1)Thr-Linker		10,5
Glc(b1-3)Glc(b1-3)[Glc(b1-6)]Glc(b1-3)Glc(b1-1)aminopentanol	*Candida spp.*	132,5
Glc(b1-3)Glc(b1-3)[Glc(b1-6)]Glc(b1-3)Glc(b1-3)Glc(b1-3)Glc(b1-3)Glc(b1-3)Glc(b1-1)aminopentanol	*Candida spp.*	236
Glc(b1-3)Glc(b1-3)[Glc(b1-6)]Glc(b1-3)Glc(b1-3)Glc(b1-3)Glc(b1-3)Glc(b1-3)Glc(b1-3)Glc(b1-3)Glc(b1-3)Glc(b1-3)Glc(b1-1)aminopentanol	*Candida spp.*	100,5

Glc(b1-3)Glc(b1-3)Glc(b1-3)Glc(b1-3)Glc(b1-3)Glc(b1-3)Glc(b1-3)Glc(b1-3)Glc(b1-3)Glc(b1-3)Glc(b1-3)Glc(b1-1)aminopentanol	*Candida spp.*	127
L-PneNAc(a1-2)GlcA(b1-3)FucNAc(a1-3)D-FucNAc(b1-1)aminopentanol	*Streptococcus pneumoniae*	24,5
Mixture of: D-6d-xylHexpNAc-4-ulo(b1-1)aminopentanol (Sugp(b1-1)aminopentanol) and D-FucNAc(b1-1)aminopentanol	*Streptococcus pneumoniae*	845,5
Mixture of: FucNAc(a1-3)D-6d-xylHexpNAc-4-ulo(b1-1)aminopentanol and FucNAc(a1-3)D-FucNAc(b1-1)aminopentanol	*Streptococcus pneumoniae*	164
FucNAc(a1-3)D-FucNAc(b1-1)aminopentanol	*Streptococcus pneumoniae*	301
GlcA(b1-4)FucNAc(a1-1)aminopentanol	*Streptococcus pneumoniae*	336
Glc(b1-3)FucNAc(a1-1)aminopentanol	*Streptococcus pneumoniae*	324,5
L-PneNAc(a1-2)GlcA(b1-1)aminopentanol	*Streptococcus pneumoniae*	670
L-PneNAc(a1-1)aminopentanol	*Streptococcus pneumoniae*	279,5
L-PneNAc(b1-1)aminopentanol	*Streptococcus pneumoniae*	276,5
Gal(b1-4)[Glc(b1-6)]GlcNAc(b1-3)Gal(b1-1)aminopentanol	*Streptococcus pneumoniae*	289,5
Glc(a1-4)Gal(a1-4)GlcA(b1-4)Glc(b1-1)aminoethanol	*Streptococcus pneumoniae*	1735,5
Glc(a1-4)Gal(a1-1)aminoethanol	*Streptococcus pneumoniae*	919,5
GlcA(b1-4)Glc(b1-4)Glc(a1-4)Gal(a1-1)aminoethanol	*Streptococcus pneumoniae*	3351
Glc(a1-4)Gal(a1-4)GlcA(b1-4)Glc(b1-1)aminopentanol	*Streptococcus pneumoniae*	836,5
Gal(a1-4)GlcA(b1-4)Glc(b1-4)Glc(a1-1)aminopentanol	*Streptococcus pneumoniae*	60

GlcA(b1-4)Glc(b1-4)Glc(a1-4)Gal(a1-1)aminopentanol	*Streptococcus pneumoniae*	567
Glc(b1-4)Glc(a1-4)Gal(a1-4)GlcA(b1-1)aminopentanol	*Streptococcus pneumoniae*	511,5
Xyl(b1-4)Xyl(b1-1)aminopentanol		642
Xyl(b1-4)Xyl(b1-4)Xyl(b1-4)Xyl(b1-1)aminopentanol		4,5
Xyl(b1-4)Xyl(b1-4)Xyl(b1-4)Xyl(b1-4)Xyl(b1-4)Xyl(b1-1)aminopentanol		0
Xyl(b1-4)Xyl(b1-4)Xyl(b1-4)Xyl(b1-4)Xyl(b1-4)Xyl(b1-4)Xyl(b1-4)Xyl(b1-1)aminopentanol		0
Glc(b1-4)Glc(b1-4)Glc(b1-4)Glc(b1-1)aminopentanol		85
Glc(b1-3)GlcA(b1-4)Glc(b1-1)aminopentanol	*Streptococcus pneumoniae*	1175
GlcA(b1-4)Glc(b1-1)aminoethanol	*Streptococcus pneumoniae*	402,5
Glc(b1-3)GlcA(b1-1)aminoethanol	*Streptococcus pneumoniae*	282,5
ManNAc(b1-3)FucNAc(a1-3)GalNAc(a1-4)Gal-2,3-pyruvate(a1-1)aminopentanol	*Streptococcus pneumoniae*	134
GlcA(b1-1)aminoethanol		1185,5
Glc(a1-4)GalNAc(b1-4)Man(a1-1)aminopentanol	*Toxoplasma gondii*	436
Glc(a1-4)GalNAc(b1-4)[Man(a1-2)Man(a1-6)]Man(a1-1)aminopentanol	*Toxoplasma gondii*	2121,5
Glc(a1-4)GalNAc(b1-4)[Man-6-PEtN(a1-2)Man(a1-6)]Man(a1-1)aminopentanol	*Toxoplasma gondii*	1237,5
GalNAc(b1-4)Man(a1-1)aminopentanol		447,5
GalNAc(b1-4)[Man-6-PEtN(a1-2)Man(a1-6)]Man(a1-1)aminopentanol		804
GalNAc(b1-4)[Man(a1-2)Man(a1-6)]Man(a1-1)aminopentanol		2487
GalNAc(b1-4)[Man-6-PEtN(a1-2)Man(a1-6)]Man-2-PEtN(a1-1)aminopentanol		784

GalNAc(b1-4)Man(a1-1)aminododecanol		163,5
GalNAc(b1-4)Man(a1-1)p-aminocyclohexanol		0
GlcA(b1-4)Glc(b1-3)GlcA(b1-1)aminoethanol	*Streptococcus pneumoniae*	19
GlcA(b1-4)Glc(b1-3)Glc(b1-4)Glc(b1-1)aminoethanol and/or Glc(b1-4)Glc(b1-3)GlcA(b1-4)Glc(b1-1)aminoethanol		60,5
Man(a1-1)aminopentanol		3367,5
GlcNAc-6-P-phosphoaminopentanol(a1-3)GlcNAc-6-P-phosphoaminopentanol(a1-2)glyceric acid	*Clostridium difficile*	283
GlcA(a1-3)Gal(a1-3)ManNAc(b1-4)Glc(b1-4)Glc(a1-1)aminopentanol	*Streptococcus pneumoniae*	459
GlcA(a1-3)Gal(a1-3)ManNAc-6-acetate(b1-4)Glc(b1-4)Glc(a1-1)aminopentanol	*Streptococcus pneumoniae*	0
GlcA(a1-3)Gal(a1-1)aminopentanol	*Streptococcus pneumoniae*	425,5
Glc(b1-4)Glc(a1-1)aminopentanol		1195,5
ManNAc(b1-4)Glc(b1-4)Glc(a1-1)aminopentanol	*Streptococcus pneumoniae*	208,5
GalNAc(b1-3)GalNAc(b1-1)aminopentanol		80
Glc(b1-4)Gal(b1-4)Glc(b1-1)aminopentanol		116,5
Rha(a1-3)[Rha(a1-3)Glc(b1-4)]Glc(a1-2)Glc(a1-1)aminopentanol	*Clostridium difficile*	893
GlcNAc(a1-3)GlcNAc-6-P-phosphoaminopentanol(a1-2)glyceric acid	*Clostridium difficile*	175
GlcNAc(a1-3)GlcNAc[(a1-2)glyceric acid](6-P-6)GlcNAc(a1-3)GlcNAc-6-P-phosphoaminopentanol(a1-2)glyceric acid	*Clostridium difficile*	1288
Man(a1-2)Man(a1-2)[Gal(b1-4)]Man(a1-1)aminoethanol	*Leishmania donovani*	1910,5
Glc(b1-3)Gal(b1-4)Man(a1-1)aminopentanol	*Leishmania chagasi*	304
Rha(a1-2)Rha(a1-2)Rha(a1-1)aminopentanol	*Klebsiella pneumoniae*	1507,5

GalNAc-2,3-Oxazolidinone(a1-4)GalNAc-2,3-Oxazolidinone(a1-1)aminopentanol		273
Glc(a1-2)Glc(a1-3)[FucNAc(a1-3)GalNAc(b1-4)]ManNAcA(b1-1)aminopentanol	*Streptococcus pneumoniae*	1078,5
Glc(a1-2)Glc(a1-3)[Gal(a1-3)FucNAc(a1-3)GalNAc(b1-4)]ManNAcA(b1-1)aminopentanol	*Streptococcus pneumoniae*	15
Araf(a1-3)[Araf(a1-5)]Araf(a1-5)Araf(a1-1)aminopentanol	*Mycobacterium tuberculosis*	1706,5
Man(a1-5)Araf(a1-3)[Man(a1-5)Araf(a1-5)]Araf(a1-5)Araf(a1-1)aminopentanol	*Mycobacterium tuberculosis*	1593,5
GalNAc-3,4-diacetate(a1-4)GalNAc-3-acetate(a1-4)GalNAc-3-acetate(a1-4)GalNAc-3-acetate(a1-1)aminopentanol		300,5

[1]Glycan and microbial origin data for this table retrieved from supplemental material in ref [27].

[2]Concanavalin A (ConA) was used as a positive control in this glycan array. Only values for ConA are shown as no signals were detected for AsCTL-42 (see Fig. S3A).
