# Supplementary figures and images for: Lectin-Mediated Bacterial Modulation by the Intestinal Nematode Ascaris suum
Source: Int J Mol Sci. 2021 Aug 14;22(16):8739. doi: 10.3390/ijms22168739 (PMC8395819; doi:10.3390/ijms22168739)

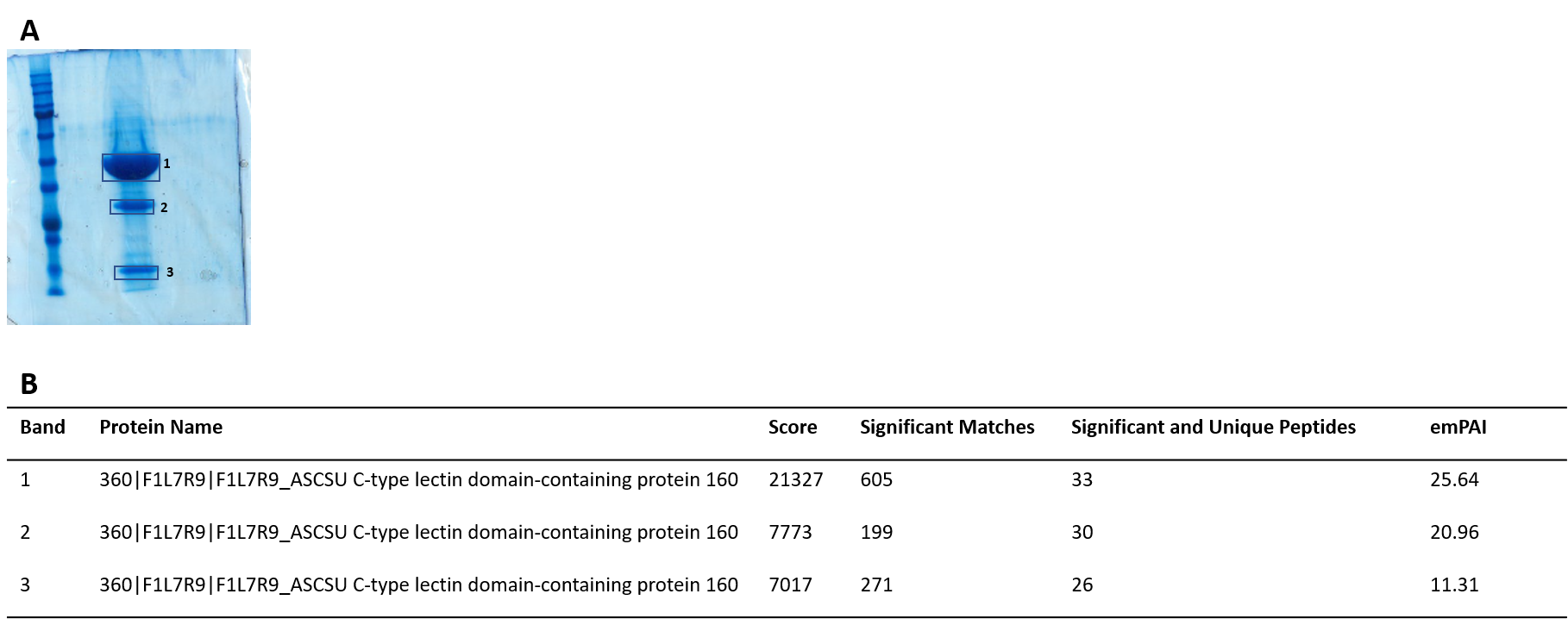

Supplement: Supplementary file 1 [file ijms-22-08739-s001.zip › Figure S1.tif]

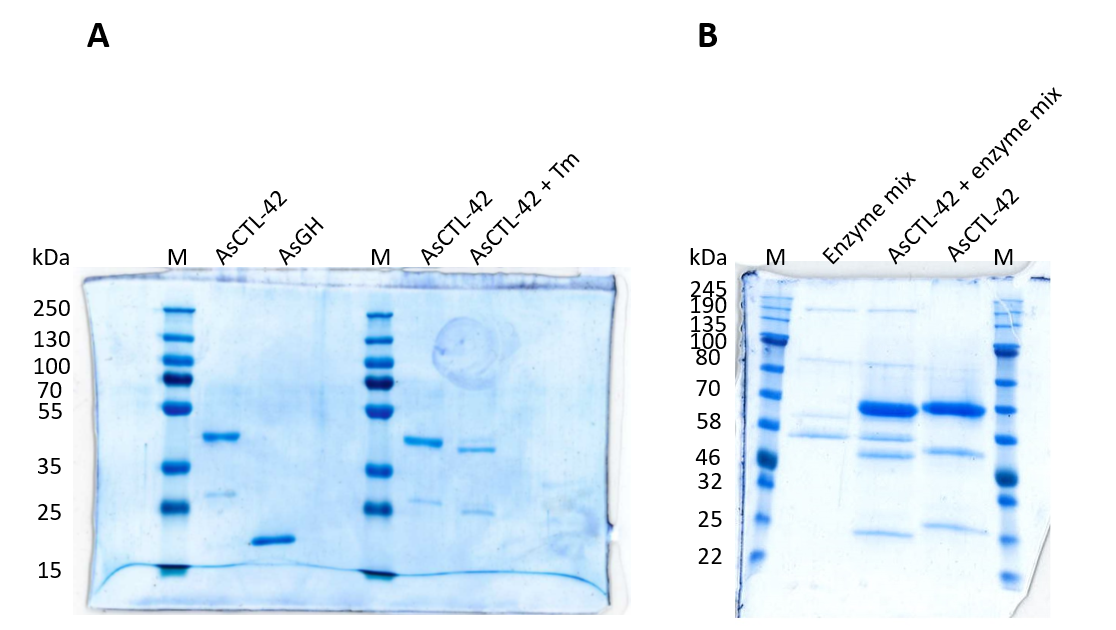

Supplement: Supplementary file 1 [file ijms-22-08739-s001.zip › Figure S2.tif]

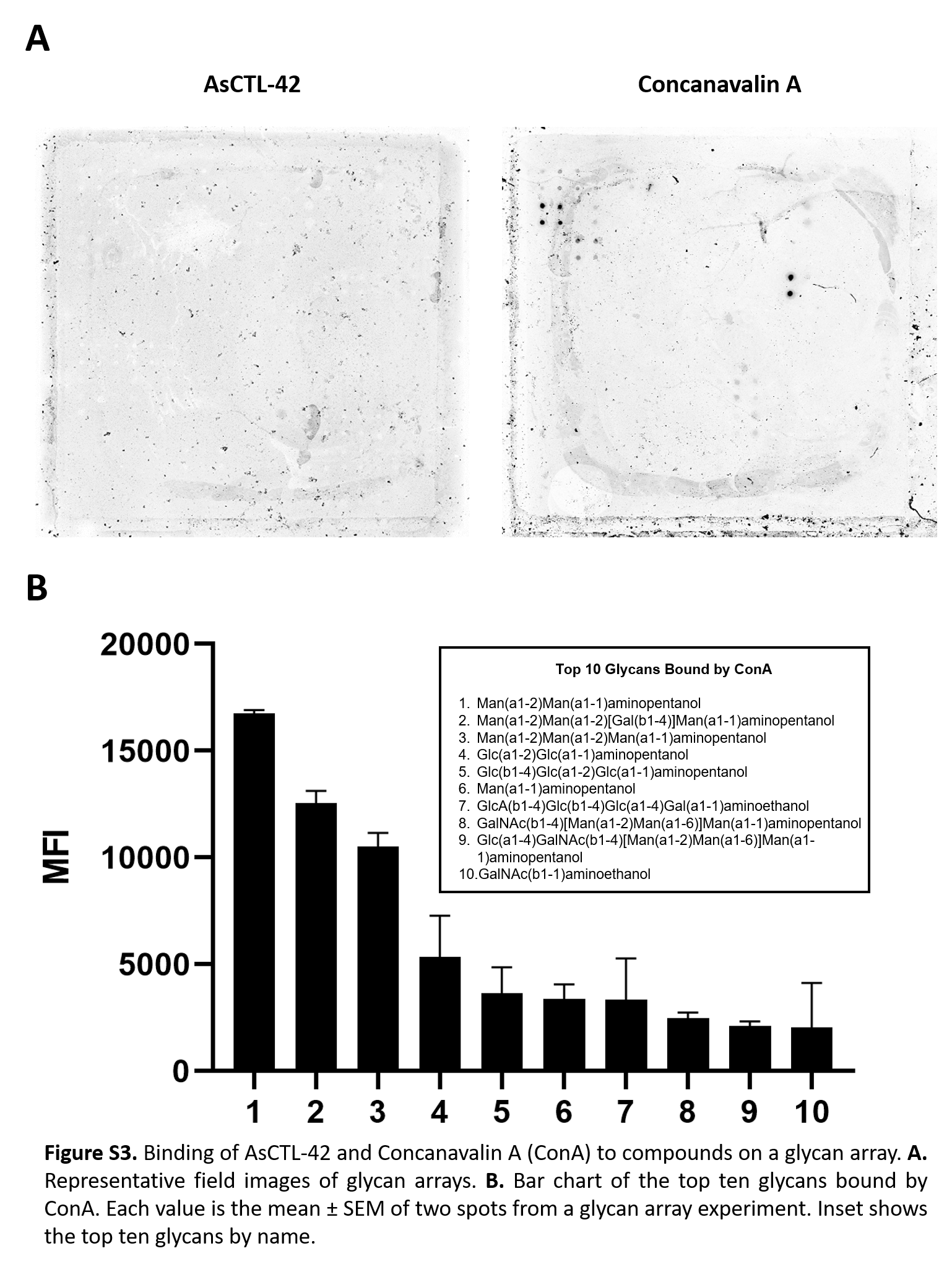

Supplement: Supplementary file 1 [file ijms-22-08739-s001.zip › Figure S3.tif]
